# Supplementary material for: Circulation of hydraulically ponded turbidity currents and the filling of continental slope minibasins
Source: Nat Commun. 2024 Mar 7;15:2075. doi: 10.1038/s41467-024-46120-2 (PMC10920650; doi:10.1038/s41467-024-46120-2)
Supplement: Supplementary file 2 — Description of Additional Supplementary Files [file 41467_2024_46120_MOESM2_ESM.pdf]

Description of Additional Supplementary Files

File Name: Supplementary Movie 1
Description: Overhead time-lapse of the first flow event in the lowflux experiment (TDWB-21-2). Video shown at 24 times actual speed with frames separated by 4 sec. Tick marks on edge of video occur every 1.0 m.

File Name: Supplementary Movie 2
Description: Overhead time-lapse of the first flow event in the midflux experiment (TDWB-21-4). Video shown at 24 times actual speed with frames separated by 4 sec. Tick marks on edge of video occur every 1.0 m.

File Name: Supplementary Movie 3
Description: Overhead time-lapse of the second flow event in the high-flux experiment (TDWB-21- 3). Video shown at 24 times actual speed with frames separated by 4 sec. Tick marks on edge of video occur every 1.0 m

File Name: Supplementary Movie 4
Description: Overhead time-lapse of all three experiments with timing of frames synced to the opening of the valve at the beginning of an experiment that initiated delivery of a slurry to the basin. Video shown at 24 times actual speed with frames separated by 4 sec. Tick marks on edge of video occur every 1.0 m.
